# Plasma Estradiol and Endometrial Edema Profile in Acyclic Mares After Single Administration of 17‐β Estradiol, Estradiol Benzoate and Estradiol Cypionate

**DOI:** 10.1111/rda.70108

**Published:** 2025-08-04

**Authors:** Laís Andrade Barbosa, Arthur Pelegi Maran, Maria Eduarda Rodrigues de Almeida, Ednaldo Carvalho Guimarães, Beatriz Bringel, Robert H. Douglas, Thereza Fornazier Good Lima, Elisa Sant'Anna Monteiro da Silva

**Affiliations:** ^1^ School of Veterinary Medicine and Animal Science Universidade Federal de Uberlândia Uberlândia MG Brazil; ^2^ School of Mathematics Universidade Federal de Uberlândia Uberlândia MG Brazil; ^3^ B.E.T. Laboratories Rio de Janeiro RJ Brazil; ^4^ B.E.T. Laboratories Lexington Kentucky USA

**Keywords:** concentration, esters, oestrogen, protocol, recipient

## Abstract

A longer endometrial exposure to estradiol before progesterone has been shown to be beneficial in cyclic and acyclic recipient mares. Therefore, the selection of an estradiol ester that promotes longer endometrial exposure to estradiol using a single administration would be advantageous when preparing acyclic mares as embryo recipients. This study investigated plasma estradiol profiles in acyclic mares after a single administration of 17‐β estradiol (17‐β), estradiol benzoate (EB) and estradiol cypionate (EC), and the correlation between plasma concentrations and endometrial edema. Fifteen non‐cyclic mares were divided into groups 17‐β (*n* = 5), EB (*n* = 5) or EC (*n* = 5), receiving a single dose of 10 mg of the respective hormone. Blood sample collections and transrectal ultrasonography were performed every 6 h from hour 0 to 12, every 12 h from 12 to 48 h, and every 24 h from 48 to 120 h after hormone administration. Five of the acyclic mares were used during the breeding season as a cyclic control. Greater median concentrations were detected using EB (38.6 pg/mL; *p* < 0.05). For 17‐β, peak concentration was observed at 6 h (29.7 pg/mL) and decreased 24 h after administration (5.9 pg/mL; *p* < 0.05). In the EC group, there was a modest peak starting from 12 h (11.7 pg/mL; *p* < 0.05), remaining relatively constant until 120 h. A more rapid increase of edema to moderate and high scores was found when using 17β estradiol, although edema scores and persistence until Day 5 were similar among the oestrogens used. A correlation between estradiol concentration and endometrial edema was only seen when using EC, and this hormone also produced the most similar concentration values to those found in natural cycling mares. Therefore, it is likely that EC would be a suitable hormone for preparing acyclic mares as embryo recipients.

## 1 Introduction

Several hormone protocols consisting of oestrogen followed by progesterone sources have been described for preparing acyclic mares as embryo recipients (Botelho et al. 2015; Rocha Filho et al. 2004; Silva et al. 2014, 2016, 2017, 2021). While progesterone administration is fairly standardised in terms of dose and administration frequency, oestrogen treatment is extremely variable, including the estradiol ester used in addition to the treatment regimen.

In cattle, oestrogen treatment regimens have been thoroughly studied and the type and dose of estradiol ester are well established within the fixed time AI protocol (Alves et al. 2021; Mapletoft et al. 2002; Sales et al. 2012). It is well known that estradiol cypionate has a longer half‐life than estradiol benzoate, which in turn has a longer half‐life than 17‐β estradiol (Burke et al. 2000; Larson and Ball 1992; Mapletoft et al. 2002; Sales et al. 2012).

In mares, the reports on oestrogen treatment are still scarce. Setoguchi and collaborators (Setoguchi et al. 2023) described the plasma estradiol profile following the administration of estradiol cypionate, estradiol benzoate and 17‐β estradiol in decreasing doses (10, 6 and 4 mg) for three consecutive days, in which blood samples were collected every 24 h. A significant peak was detected 24 h after the first administration (D1) of estradiol benzoate, which was followed by a sharp decline on D3. In the estradiol cypionate group, the peak was observed 48 h after administration (D2), which was maintained until D4. As for the 17‐β estradiol group, a distinct peak was not observed in a 24 h blood sampling assessment, although increased edema was observed from D1 to D4. According to previous reports on single 17‐beta estradiol administration in ovariectomized cows, it is likely that the peak occurs earlier (Setoguchi et al. 2023).

Moreover, longer endometrial exposure to estradiol before ovulation or progesterone administration has been shown to be beneficial for pregnancy rates and uterine environment in cyclic and acyclic treated recipient mares (Cuervo‐Arango et al. 2018; Silva et al. 2019, 2021, 2024). Therefore, the selection of an estradiol ester that promotes longer endometrial exposure to estradiol using a single administration would be advantageous for field conditions when preparing acyclic mares as embryo recipients.

Therefore, this study aimed to assess plasma estradiol profiles and uterine oedema after a single administration of 17‐β estradiol, estradiol benzoate or estradiol cypionate. More frequent blood collections were made during the first 48 h after oestrogen administration, in order to detect peak concentrations more accurately. The uterine oedema persistence was also investigated and correlated to estradiol concentrations. In addition, estradiol concentrations and oedema scores were obtained from cyclic mares to serve as a physiological control group.

## 2 Material and Methods

### 2.1 Animals

Fifteen crossbred mares, between 3 and 14 years of age, and weighing between 350 and 450 kg, were used for this study. Mares were kept in paddocks with free access to african star grass pasture ( *Cynodon plectostachyus* ), water and mineralized salt, which belonged to the research and teaching herd of the Federal University of Uberlândia—Minas Gerais—Brazil. The experiment was conducted from July to December 2023 and all animal procedures were approved by the Ethics Committee on the Use of Animals (CEUA) of the Federal University of Uberlândia (n° 23117.021317/2024‐53).

Only mares in seasonal anestrus were selected for the acyclic treated groups, which showed absence of a corpus luteum (CL), endometrial oedema, uterine tone and the presence of follicles ≤ 20 mm for at least 21 consecutive days. In addition, plasma P4 concentrations below 1 ng/mL were observed in all mares prior to treatment initiation (mean 0.34 ng/mL, range 0.05–0.83 ng/mL).

As for the cyclic group, the same mares used during the acyclic phase were monitored on a weekly basis for follicular activity and ovulation. Data was collected starting from the second cycle of the breeding season. Five mares out of the 15 were included in the cyclic group, in which the mares showing estrus duration of 5–6 days (with endometrial edema ≥ 1.5) were selected.

### 2.2 Hormonal Treatments

**FIGURE 1:**
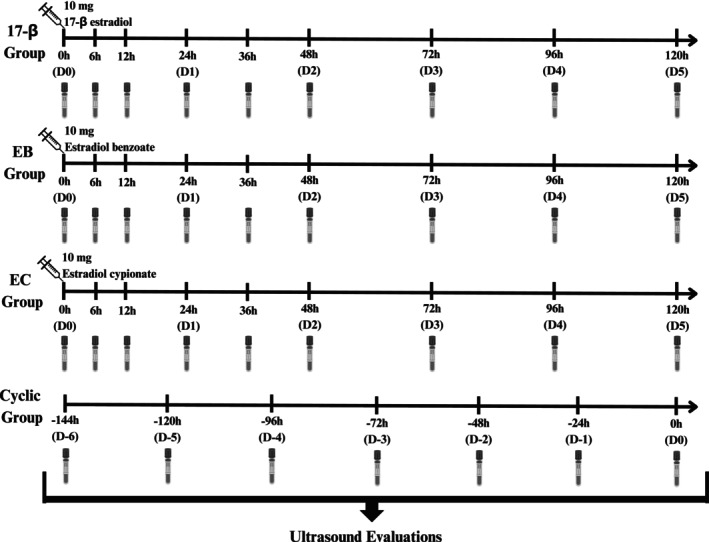
Experimental design representation including the treatment groups in anestrous mares (17‐β, 17‐beta estradiol, *n* = 5; EB, Estradiol Benzoate, *n* = 5; EC, Estradiol Cypionate, *n* = 5) and the cyclic mares group (*n* = 5). Each slash indicates endometrial edema assessment and a blood sample collection. Hour zero (0 h) within day zero (D0) was considered the moment immediately before hormone administration in the acyclic groups, whereas D0 was considered the day of ovulation in the cyclic group.

The acyclic mares were randomly divided into three treatment groups: estradiol benzoate (EB; RIC‐BE 1 mg/mL, Agener União, Brazil; *n* = 5), estradiol cypionate (EC; E.C.P. 2 mg/mL, Zoetis, Brazil; *n* = 5) and 17‐β estradiol (17‐β; 17 βeta 10 mg/mL, Botupharma, Brazil; *n* = 5), in which each mare received, intramuscularly, a single dose of 10 mg of EB, EC or 17‐β on the first day of treatment (D0), respectively. The cyclic group did not receive any hormonal treatment (Figure 1). During hormonal treatment and data collection, no significant follicular growth was observed in all acyclic mares.

### 2.3 Edema Ultrasound Evaluations

**FIGURE 2:**
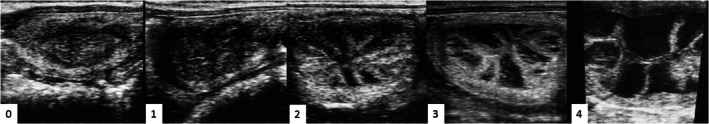
Representative ultrasound images of the mare endometrial edema, which were scored as: 0, lack of edema; 1, low edema; 2, moderate edema; 3, high edema; 4, exaggerated edema, as previously reported (Setoguchi et al. 2023).

Transrectal palpations and ultrasound evaluations for endometrial edema assessment were conducted immediately before the first hormone administration (0 h) until 5 days after (120 h; Figure 1). The endometrial edema was scored from 0 to 4, as previously reported (Setoguchi et al. 2023), in which: 0 = lack of edema; 1 = low edema; 2 = moderate edema; 3 = high edema; and; 4 = exaggerated edema (Figure 2), using 0.5 graduation intervals when deemed appropriate, depending on the intensity. Edema monitoring occurred every 6 h for the first 12 h after the oestrogen administration (0, 6 and 12 h), every 12 h until 48 h (24, 36 and 48 h), and every 24 h until Day 5 (72, 96 and 120 h; Figure 1).

For the cyclic group, mares were evaluated using transrectal ultrasound once a week to monitor follicular growth and deviation. Once a dominant follicle associated with an edema ≥ 1.5 was detected, mares were monitored on a daily basis (Figure 1) until ovulation (D0) to assess follicular diameter and edema score.

### 2.4 Blood Samples Collection, Progesterone and Estradiol Assays

Concomitantly with the transrectal palpations, including cyclic and acyclic groups, blood samples were collected via jugular vein puncture into EDTA tubes (Figure 1). Afterwards, samples were centrifuged (900 × g/10 min) and the plasma stored at −20°C until assayed. Progesterone quantifications were performed on single measurements, on the hour 0 samples from the acyclic mares, which corresponded to the moment immediately before oestrogen administration, using a radioimmunoassay commercial kit (RIA Progestreone—Beckman Coulter, Prague 10, Czech Republic). Progesterone assessment was performed to confirm the anoestrus status of the acyclic mares.

As for 17‐β estradiol quantifications, radioimmunoassay commercial kits were also used (RIA Progesterone and Ultra‐Sensitive Estradiol RIA—Beckman Coulter, Prague 10, Czech Republic). All samples were tested in a single assay and the intra‐assay coefficient of variation was 10.56%, over a range of 0.01 to 55.35 pg/mL.

### 2.5 Statistical Analysis

The assumption of residual normality for the ANOVA mathematical model was assessed using the Shapiro–Wilk test at a 5% significance level. Normality was not detected in either the estradiol concentration original data or transformed data (square root (X) or log(X)). Therefore, a non‐parametric test was used. For plasma estradiol concentrations and edema comparisons between days within each group (dependent samples), the Friedman test was used, and for comparisons between groups within each day, the Kruskal–Wallis test was applied (independent samples). In both cases, when a *p*‐value < 0.05 was found, Dunn's multiple comparisons test based on mean ranks was employed to identify the differences. Data were presented as median scores and quartiles. The Spearman test was used to determine the correlation between plasma concentrations and edema scores. All statistical tests were conducted via the open access software R (R Project 2021). Statistical difference was considered when *p* ≤ 0.05.

## 3 Results

### 3.1 Endometrial Edema

**FIGURE 3:**
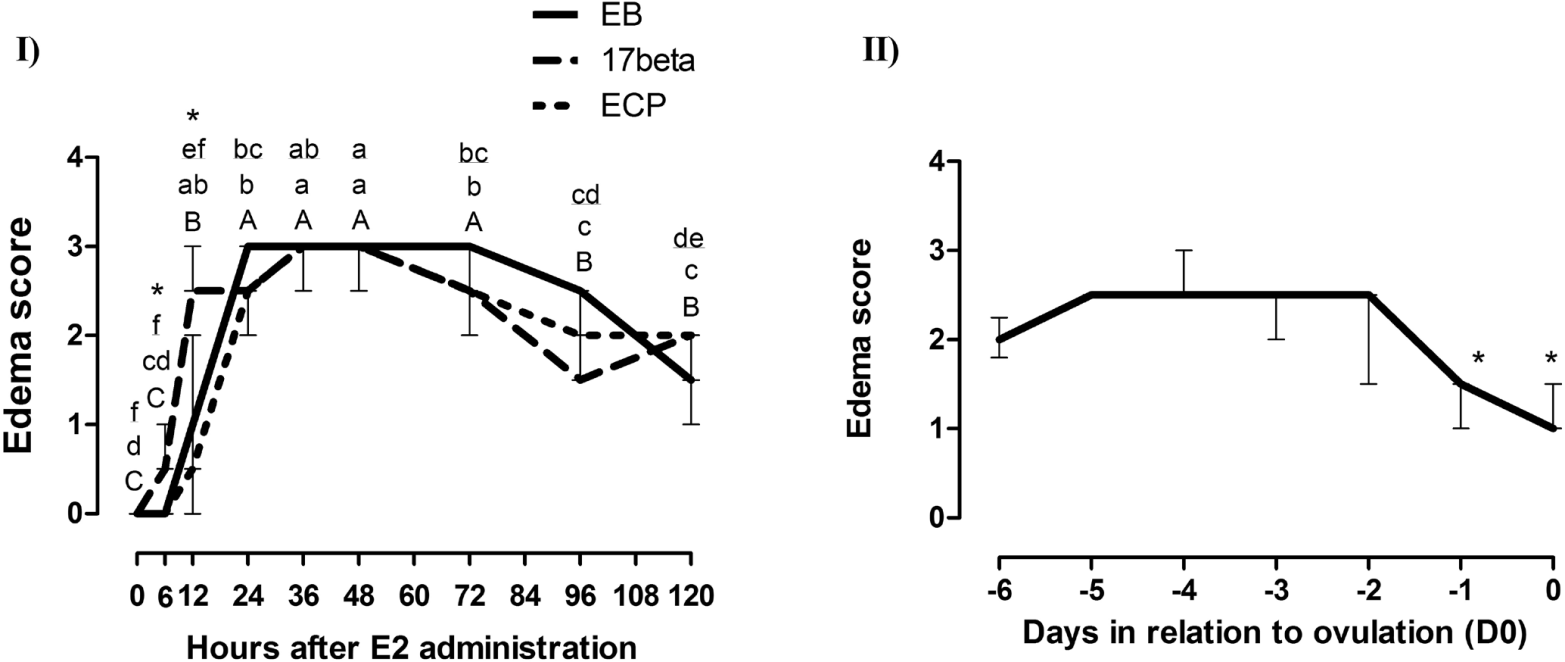
Median endometrial edema scores, with superior and inferior quartiles, in (I) anestrous mares, after administration of 10 mg of 17‐β estradiol (group 17‐β, *n* = 5), Estradiol Benzoate (group EB, *n* = 5) and Estradiol Cypionate (group EC, *n* = 5); and in (II) non‐treated cyclic mares (*n* = 5), during natural estrus, starting from 6 days prior to ovulation (D0). *Asterisks represent differences between groups. Different capital letters show differences between hours in the EB group; different lowercase letters show differences between hours in the 17‐β group; and different underlined letters show differences between hours in the EC group. Significance was set at *p* ≤ 0.05.

Median edema scores for acyclic groups are shown in Figure 3I. In the 17β group, the first significant increase was seen 12 h after E2 administration, which was considered a moderate to high edema (median 2.5, min = 2.5, max = 3; *p* < 0.05). The decrease was observed 72 h after and reached lower scores (< 2) subsequently (*p* < 0.05). In the EB group, despite a significant increase at 12 h past E2 administration (median 2, min = 0.5, max = 2; *p* < 0.05), maximum edema was reached 24 h (D1) after the hormone was given (median 3, min = 3, max = 3; *p* < 0.05), which was maintained until D3 (72h), decreasing gradually to a low score on D5 (120h). In the EC group, a significant increase was detected (*p* < 0.05) on D1 (24h, median 2.5, min = 2, max = 2.5), followed by a subsequent increase on D2 (48h, median 3, min = 3, max = 3) and a decrease on D3 (median 2.5, min = 2.5, max = 2.5; *p* < 0.05). A gradual decrease was observed thereafter and the median edema score was moderate until the last assessment on D5 (score 2). When comparisons were made between groups, a greater edema score was observed at 6 h (median 0.5, min = 0.5, max = 1.0) and 12 h (median 2.5, min = 2.5, max = 3) in group 17β when compared to EB and EC groups (*p* < 0.05). There were no differences between groups on the remaining days (*p* > 0.05).

For the cyclic group, median edema scores are shown in Figure 3II. Median edema score was moderate to high from D‐6 to 48 h prior to ovulation (D‐2). A significant reduction was observed on D‐1 (median 1.5, min = 1, max = 1.5) and D0 (median 1.0, min = 1.0, max = 1.5; *p* < 0.05).

### 3.2 Estradiol Concentrations

**FIGURE 4:**
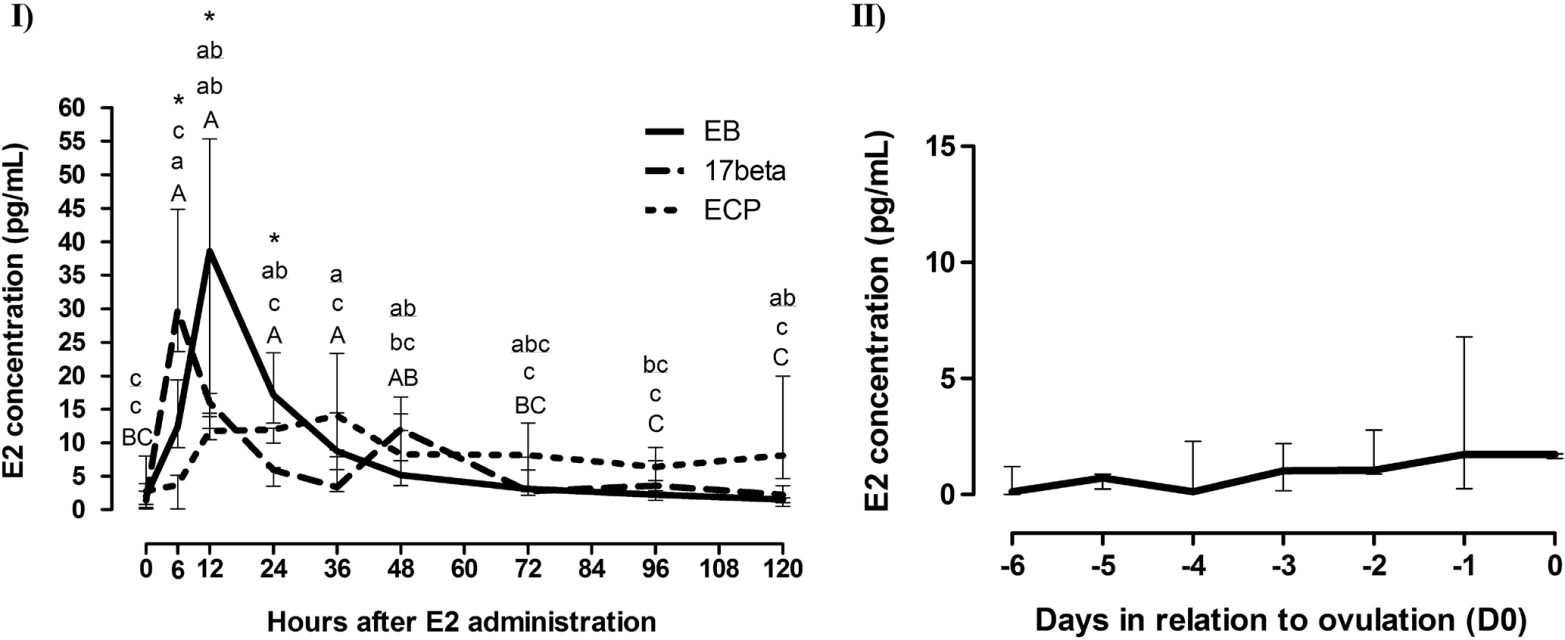
Median plasma estradiol (E2) concentrations, with superior and inferior quartiles, in (I) anestrous mares after administration of 10 mg of 17‐β estradiol (group 17‐β, *n* = 5), Estradiol Benzoate (group EB, *n* = 5) and Estradiol Cypionate (group EC, *n* = 5); and in (II) non‐treated cyclic mares (*n* = 5), during natural estrus, starting from 6 days prior to ovulation (D0). *Asterisks represent diferences between groups within each hour. Different capital letters show differences between hours in the EB group; different lowercase letters show differences between hours in the 17‐β group; and different underlined letters show differences between hours in the EC group. Significance was set at *p* ≤ 0.05.

Median plasma estradiol concentrations for the acyclic groups are shown in Figure 4I. Maximum values were observed at 6, 12 and 36 h in groups 17‐β (median 29.7 pg/mL, min = 23.6, max = 44.9), EB (median 38.6 pg/mL, min = 12.1, max = 55.3) and EC (median 14 pg/mL, min = 8.9, max = 14.4), respectively. After E2 administration, an increase was detected at 6 h for group 17‐β (median 29.7 pg/mL, min = 23.6, max = 44.9; *p* < 0.05), followed by a decrease from D1 (24h, median 5.9 pg/mL, min = 3.5, max = 6.3) onwards (*p* < 0.05). As for EB, an increase was also observed at 6 h (median 12.4 pg/mL, min = 9.2, max = 19.4, *p* < 0.05), although a decrease was only detected from D2 onwards (*p* < 0.05) when compared to hour 12 (maximum value). With regards to the EC group, an increase was observed at 12 h (median 11.7 pg/mL, min = 10.5, max = 13.9; *p* < 0.05), and a decrease after D4 (96h, *p* < 0.05). At 6 h after E2 administration, plasma concentrations were greater in 17‐β than in the EB group (29.7 pg/mL and 12.4 pg/mL, respectively), which in turn was higher than in the EC group (3.67 pg/mL; *p* < 0.05). At 12 h, concentrations were higher in EB than in the EC group (38.6 pg/mL and 11.7 pg/mL, respectively; *p* < 0.05), although not different from 17‐β (16 pg/mL; *p* < 0.05). At 24 h, plasma concentration was highest in the EB group (median 17.1 pg/mL, min = 12.9, max = 23.4; *p* < 0.05).

As for the cyclic group, median plasma estradiol concentrations are shown in Figure 4II. Maximum values were observed on D‐1 (median 1.73 pg/mL, min = 0.25, max = 6.8) and D0 (median 1.73 pg/mL, min = 1.56, max = 1.76); however, no significant differences were detected between days (*p* > 0.05).

A significant positive correlation between plasma E2 concentrations and endometrial edema was only detected in the EC group (*p* = 0.005; *r* = 0.4). For the remaining groups, the coefficients of correlation were as follows: EB group (*p* = 0.54; *r* = 0.09), 17β group (*p* = 0.40; *r* = −0.10) and Cyclic group (*p* = 0.4; *r* = −0.14).

## 4 Discussion

A more frequent assessment of E2 plasma concentrations, in the first 48 h after a single administration of 10 mg of 17‐β estradiol, showed that a significant increase and decrease occur within the first 24 h. In addition, highest E2 concentrations are achieved after EB administration, while EC produces a modest increase, which remains relatively constant for a longer period of time.

In a previous study also performed in acyclic mares by our research group, a peak in plasma E2 concentration was not detected in a 24 h assessment after 17‐β estradiol was given (Setoguchi et al. 2023). In ovariectomized cows treated with a single dose of 5 mg of 17‐beta, the peak was detected at 12 h after administration (Martínez et al. 2005). Therefore, we hypothesised that the peak could also have occurred earlier in mares. The present results have confirmed our hypothesis, in which the peak value was detected by 6 h after 17‐β estradiol was given and, as in cows, the concentration had significantly reduced by 24 h. However, the edema score was not associated with the estradiol profile, remaining moderate to high from 12 to 72 h. Therefore, no correlation was detected between E2 concentration and edema score in the 17‐β group, while a weak correlation was seen in our previous study (Setoguchi et al. 2023), likely because of the additional doses administered.

Compared to the other esters, EB produced the highest E2 concentration peak. This finding is in agreement with the previous study conducted in acyclic mares, in which the highest E2 concentration was also obtained using EB, although at 24 h, which was the first blood sampling after the hormone administration (Setoguchi et al. 2023). Herein, the first significant increase was detected at 6 h, although the highest numerical concentration was at 12 h. An earlier decrease was expected at 48 h, since only a single dose was administered. While increased E2 concentration lasted from 6 to 48 h, endometrial edema remained high from 24 to 72 h and gradually decreased to a low score at 120 h. Thus, unlike previously reported (Setoguchi et al. 2023), a correlation was not observed in this study, probably due to the single administration and to the longer lasting effects of bound oestrogen to endometrial receptors.

As described in cows (Vynckier et al. 1990) and mares (Setoguchi et al. 2023), the lowest peak concentration was observed after EC administration. A significant increase occurred at 12 h, and the concentrations remained relatively constant until 120 h. Although lower concentrations were detected in the present study, the EC profile was similar to that found in acyclic mares receiving a total of 20 mg in decreasing doses for three consecutive days (Setoguchi et al. 2023). In addition, the concentration values after EC administration were the closest to those observed in cyclic mares. Regarding endometrial edema, the first increase was seen at 24 h and the highest scores at 36 and 48 h. The EC group was the only one that showed a positive, although weak, correlation between edema scores and plasma estradiol concentration when administering a single dose.

Furthermore, in the present study we have included cyclic mares as a physiological control. Plasma E2 concentrations in cyclic mares start to increase 8–6 days prior to ovulation and peak values occur around 24 to 48 h before (Gastal et al. 1999; Ginther 1992; Shirazi et al. 2003). In the present study, a significant peak was not observed, although the maximum median values were detected at 24 h prior to ovulation (D‐1). In cyclic mares, great individual variances in plasma estradiol can be expected (Daels et al. 1991; Gastal et al. 1999; Ginther 1992; Meinecke et al. 1987; Shirazi et al. 2003) and, associated with the limited sample size in the cyclic group, the lack of significance between hours within the group could be explained. In addition, it is also possible that differences in assay methodology (i.e., use of ether extraction or commercial kits) could account for a lack of a significant peak of estradiol before ovulation in this study. Nonetheless, the inclusion of cyclic mares as controls was relevant to compare plasma profiles and concentration values to those obtained in the acyclic ones treated with the different estradiol esters, in order to see if there was a most similar one, which apparently was the EC group. As for endometrial edema, a moderate to high score was observed until D‐2, with a significant decrease until ovulation (D0), which is in agreement with several previous reports (Ginther 1992; Ginther and Pierson 1984; Pelehach et al. 2002; Samper 1997). Therefore, no correlation between uterine edema and plasma E2 concentration was detected in the cyclic group, as also described in early reports by Pycock et al. and Watson et al. (Pycock et al. 1995; Watson et al. 2003).

The different profiles observed after using the three estradiol esters are expected considering the differences in their molecules. Estradiol esters are synthesised by esterification of the 17β‐hydroxyl group of 17β‐estradiol, resulting in a prolonged pharmacological effect due to increased resistance of the molecule to metabolic degradation. The half‐life of each compound is influenced by the polarity of the molecule, which is determined by its molecular size, as well as by the presence of aromatic rings and the symmetry of its chemical bonds (Mapletoft et al. 2002). Estradiol cypionate is synthesised through the esterification of estradiol with cyclopentylpropionic acid and exhibits a more prolonged effect compared to 17β‐estradiol and estradiol benzoate. In contrast, estradiol benzoate is formed by esterification at carbon 3 and has a shorter duration of action than estradiol cypionate (Mapletoft et al. 2002), although it still provides a longer effect than 17β‐estradiol, as the latter does not require metabolic conversion to reach its biologically active form (Larson and Ball 1992).

As a limitation of the study, it is important to point out that the exogenous estradiol esters used were from different companies and that the type of vehicle in each product was not disclosed. Even though it is known that these esters have different half‐lives (Burke et al. 2000; Larson and Ball 1992; Mapletoft et al. 2002; Sales et al. 2012), the vehicle in which they are formulated can also interfere with its metabolisation and plasmatic release.

Since a single administration of EC increased E2 concentrations and maintained endometrial edema at a moderate to high score for at least 5 days, we believe it would be a suitable hormone for preparing acyclic mares as embryo recipients, when considering less animal handling in addition to providing a better uterine environment. Previous studies in acyclic and cyclic mares have shown that a longer exposure to estradiol before progesterone may benefit the uterine environment in terms of greater expression of genes involved in uterine receptivity (Silva et al. 2019, 2021, 2024), as well as increased pregnancy rates in cyclic recipients (Cuervo‐Arango et al. 2018). However, there is still no controlled study on pregnancy rates in acyclic recipient mares exposed to a single dose of EC for more than 3 days before progesterone administration. Moreover, it is unknown whether the estradiol concentration or the presence and duration of obvious endometrial edema is more important for endometrial receptivity and therefore embryo survival.

In conclusion, through more frequent blood sampling, different plasma estradiol peaks and profiles were confirmed between 17β estradiol, estradiol benzoate, and estradiol cypionate. A significant increase and decrease occur within the first 24 h after 17β estradiol administration. In addition, highest E2 concentrations are achieved after EB administration, while EC produces a modest increase, which remains relatively constant for a longer period of time. There is a more rapid increase of edema to moderate and high scores when using 17β estradiol, although edema scores and persistence until Day 5 are similar among the three oestrogen esters used. Regarding correlation between E2 concentrations and edema scores, a weak correlation was detected only when EC was used. Moreover, the closest E2 plasma concentration values to the control group were observed when using EC as the oestrogen source. These findings provide valuable insights for optimising equine reproductive hormone protocols.

## Author Contributions

**Laís Andrade Barbosa:** writing – original draft, data collection, formal analysis. **Arthur Pelegi Maran:** investigation, data collection. **Maria Eduarda Rodrigues de Almeida:** data collection. **Ednaldo Carvalho Guimarães:** statistical analysis. **Beatriz Bringel:** investigation, validation, writing – review and editing. **Robert H. Douglas:** investigation, validation, writing – review and editing. **Thereza Fornazier Good Lima:** validation, supervision. **Elisa Sant'Anna Monteiro da Silva:** experimental design, manuscript writing, investigation, formal analysis, review and editing.

## Conflicts of Interest

The authors declare no conflicts of interest.

## Data Availability

The data that support the findings of this study are available from the corresponding author upon reasonable request.
